# Antiplatelet therapy for elderly patients with Acute Coronary Syndromes

**DOI:** 10.18632/aging.101553

**Published:** 2018-09-11

**Authors:** Stefano Savonitto, Nuccia Morici, Stefano De Servi

**Affiliations:** 1 Manzoni Hospital, Lecco, Italy; 2 Niguarda Hospital, Milano, Italy; 3 IRCCS Multimedica, Sesto San Giovanni, Italy

**Keywords:** acute coronary syndrome, elderly, antithrombotic therapy

**Figure 1:**
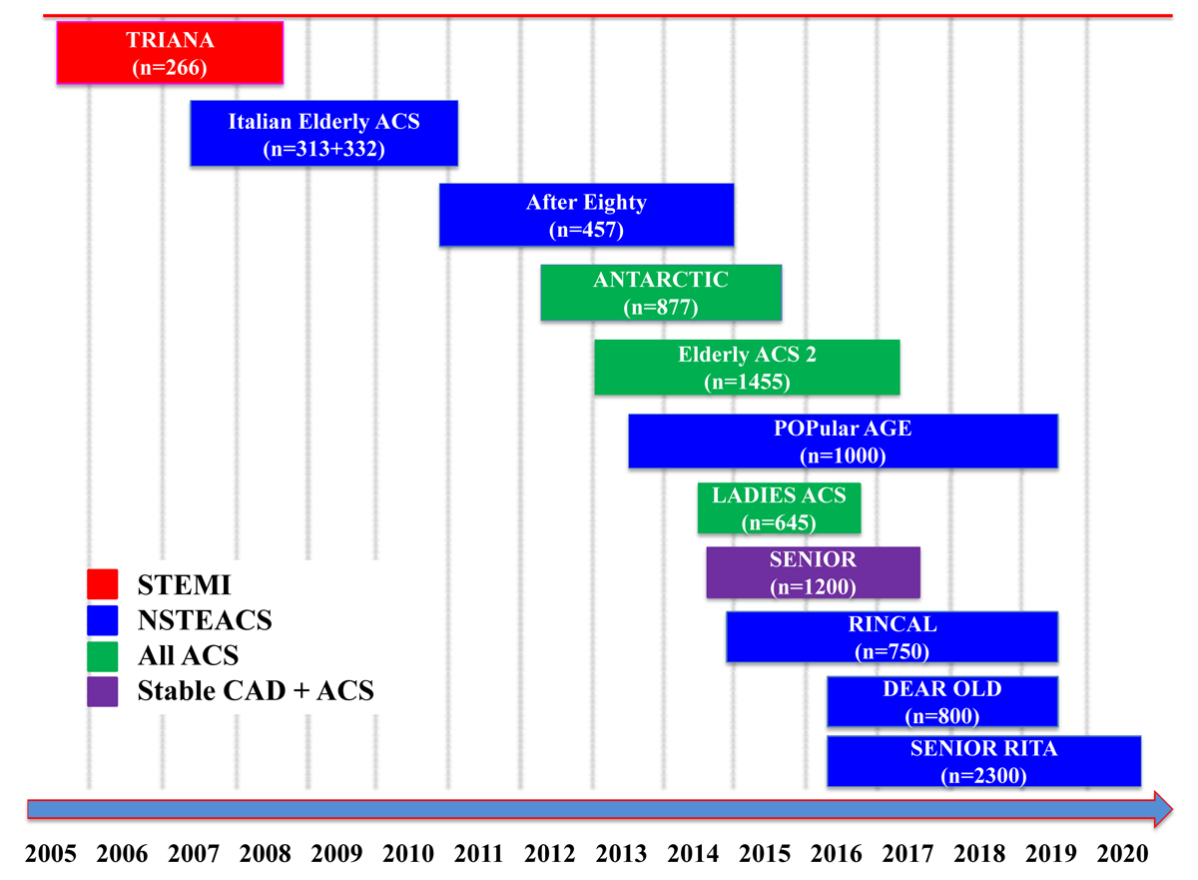
**Elderly-specific prospective trials in patients with Acute Coronary Syndromes.** STEMI: ST-Elevation Myocardial Infarction. NSTEACS: Non ST Elevation Acute Coronary Syndrome. CAD: Coronary Artery Disease.

Elderly patients represent more than one third of Coronary Care Unit admissions for Acute Coronary Syndromes (ACS). Until just a few years ago, they were largely under-represented in randomised controlled trials (RCT) forming the evidence base of practice guidelines. Over the last 10 years, specific RCTs have been carried on in this population (Figure 1), showing an overall benefit from early revascularization by percutaneous coronary intervention (PCI) both in patients with ST-elevation and in those with non-ST-elevation ACS. However, whereas elderly patients are at high risk of death and recurrent ischemic events after an ACS, they are also at high risk of bleeding complications from antithrombotic medications used to prevent such events.

Antiplatelet therapy after an ACS has been clearly shown to reduce the risk of re-infarction and stent thrombosis. This benefit was first shown with low-dose (75-100 mg daily) aspirin, and then combining an inhibitor of the platelet ADP P2Y_12_ receptor, clopidogrel, in the so called dual antiplatelet therapy (DAPT), which is currently guideline-recommended for at least 12 months after an ACS, irrespective of patient age [1]. However, there is no exception to the rule that the higher the number of drugs and their antithrombotic power, and the longer the duration of therapy, the higher is the risk of bleeding complications, even fatal, particularly in the elderly. This fact became clear for the use of even low-dose aspirin [2], and has been confirmed with DAPT combining aspirin with clopidogrel or one of the two more potent P2Y_12_ receptor blockers, prasugrel or ticagrelor. As compared to clopidogrel, the latter two agents have been shown to increase the rate of major bleeding complications by about 30%, without providing additional benefit in the elderly.

Recent RCTs have investigated strategies to tailor the power of P2Y_12_ blockade in patients aged >75 years, either by adapting in the individual patient the dose of prasugrel to the level of ADP receptor blockade measured by platelet function testing [3]; or by using reduced-dose prasugrel (5 mg, instead of the standard 10 mg once daily) [4]. However, both strategies have been unsuccessful in improving the risk vs benefit ratio of DAPT, showing neither reduction in bleeding nor better prevention of recurrent ischemic events. An alternative antithrombotic approach being tested in a new series of RCTs is the so called “aspirin-free” strategy using a single P2Y_12_ receptor blocker, ticagrelor, after a single month of DAPT [5]. The rationale for this strategy should be that aspirin is the main culprit for bleeding, particularly gastro-enteric, whereas other antithrombotic agents used in combination only potentiate aspirin bleeding risk. However, in the first of these trials using full-dose ticagrelor monotherapy for 24 months after only one month of DAPT, this strategy failed to show superiority in terms of better ischemic protection or lower bleeding, as compared to standard, guideline-recommended 12-month DAPT followed by aspirin monotherapy [6].

A simpler strategy to be tested might be shortening the duration of DAPT to one to three months (the initial period of stent thrombosis risk after an ACS), and then going on indefinitely with single antiplatelet therapy using the inexpensive low-dose aspirin. As a matter of fact, the recommendation for 12-month DAPT has been based on the results of a single trial [7] carried on in the latest years of the 20^th^ century, and only in patients with NSTEACS. Since that time, a number of advances have been made in terms of much safer stent technology, much increased operator expertize in terms of PCI, and concomitant drug therapy after an ACS, suffice it to mention the systematic use of statins and betablockers.

Recommendations on the optimal combination and duration of antiplatelet and anticoagulant therapy for (mostly elderly) patients with an ACS and atrial fibrillation are based on disputable evidence, since all the concluded and ongoing RCTs have been powered for safety only, thus leaving to clinicians an individual assessment of risk vs benefit. According to current recommendations [8], triple antithrombotic therapy using aspirin, clopidogrel and a direct anticoagulant should be kept to a minimum length of time, considering both the ischemic risk and the bleeding risk. Treatment strategies span from using only clopidogrel and an oral direct anticoagulant (in patients at very high risk of bleeding and moderate ischemic risk), to a strategy of triple antithrombotic therapy for 3-6 months followed by clopidogrel and the direct oral anticoagulant in cases at high ischemic risk and moderate bleeding risk. In any case, long term treatment without antiplatelet therapy is recommended using oral anticoagulation. Also this latter recommendation is not based on formal evidence.
